# Systematic identification and characterization of long noncoding RNAs (lncRNAs) during *Aedes albopictus* development

**DOI:** 10.1371/journal.pntd.0010245

**Published:** 2022-04-13

**Authors:** Wenjuan Liu, Peng Cheng, Kexin Zhang, Maoqing Gong, Zhong Zhang, Ruiling Zhang

**Affiliations:** 1 Collaborative Innovation Center for the Origin and Control of Emerging Infectious Diseases, Shandong First Medical University (Shandong Academy of Medical Sciences), Tai’an, China; 2 School of Basic Medical Science, Shandong First Medical University (Shandong Academy of Medical Sciences), Tai’an, China; 3 Shandong Institute of Parasitic Diseases, Shandong First Medical University (Shandong Academy of Medical Sciences), Jining, China; Connecticut Agricultural Experiment Station, UNITED STATES

## Abstract

**Background:**

*Aedes albopictus* originated in the tropical forests of Southeast Asia and can currently be found on all continents. As one of the main arboviral vectors, the control of *Ae*. *albopictus* requires novel strategies, informed by a deep knowledge of its biology. Little is known regarding mosquito long noncoding RNAs (lncRNAs), which are transcripts longer than 200 nucleotides that lack protein-coding potential and have roles in developmental regulation.

**Results:**

Based on RNA-seq data from five developmental time points, eggs, early larvae, late larvae, pupae, and adults (female and male) of *Ae*. *albopictus*, 21,414 lncRNAs were characterized in this study. Differential expression analysis revealed that lncRNAs exhibited developmental stage specificity. The expression of most lncRNAs was upregulated at the onset of metamorphosis developmental stages. More differentially expressed lncRNAs were observed between eggs and early larvae. Weighted gene co-expression network analysis (WGCNA) further confirmed that the expression patterns of lncRNAs were obviously correlated with specific developmental time points. Functional annotation using co-expression analysis revealed that lncRNAs may be involved in the regulation of metamorphic developmental transitions of *Ae*. *albopictus*. The hub lncRNAs and hub gene clusters were identified for each module that were highly associated with specific developmental time points.

**Conclusions:**

The results of this study will facilitate future researches to elucidate the regulatory mechanisms of lncRNAs in the development of *Ae*. *albopictus* and utilize lncRNAs to assist with mosquito control.

## Author summary

*Aedes albopictus* is an invasive vector mosquito associated with substantial public health concerns. It is a vector of dengue, chikungunya, and Zika viruses. High levels of ecological and physiological plasticity promote rapid global invasion. In the absence of effective vaccines or specific antiviral drugs for the majority of arbovirus diseases, vector control is based mostly on chemical insecticides. However, the extensive and intensive use of chemical insecticides for mosquito control has caused several environmental and human health concerns. As holometabolous insects, mosquitoes undergo considerable structural and functional remodeling during the metamorphosis. Long noncoding RNAs (lncRNAs) have been reported to play roles in developmental regulation. To investigate the expression profiles of lncRNAs across the life cycle of *Ae*. *albopictus*, five developmental time points, eggs, early larvae, late larvae, pupae, and adults (female and male), covering all developmental stages were included in this study. Differences in lncRNA expression patterns and hub lncRNAs that were highly associated with specific developmental time points were identified. Hub lncRNAs could be used as targets to block the metamorphic process and facilitate the development of efficient and sustainable mosquito control strategies.

## Introduction

The Asian tiger mosquito *Aedes albopictus* originated in the tropical forests of Southeast Asia [1]. Due to its invasiveness and aggressive spread, *Ae*. *albopictus* has spread from its native location to every continent except Antarctica over the past 40 years [1,2]. It is currently considered the most invasive mosquito species worldwide [3]. The global expansion of *Ae*. *albopictus* is largely a result of the long distances across continental international trades. Moreover, ecological plasticity is another important factor that has guaranteed the successful colonization and adaptation of this species to new environments [4,5]. *Ae*. *albopictus* in subtropical and temperate regions can produce diapausing eggs to survive at cold temperatures and pupae and adult females can undergo photoperiodic diapause to endure unfavorable environmental conditions [6,7].

Compared with *Aedes aegypti*, the most important vector of dengue virus (DENV), *Ae*. *albopictus* is considered as a less efficient vector of DENV [4,8,9]. However, *Ae*. *albopictus* can more easily adapt to new habitats and cause dengue outbreaks in areas where *Ae*. *aegypti* is not present, making it an important vector of DENV. In addition to DENV, *Ae*. *albopictus* is capable of transmitting at least 22 arboviruses, including yellow fever, chikungunya, West Nile and Zika viruses [8,10]. As a consequence of its rapid global expansion and high adaptation capacity, this aggressive human-biting mosquito is becoming more important in the epidemic of dengue and creating new opportunities for other arboviruses [9].

Despite many studies, there are currently no effective vaccines or treatments for most *Ae*. *albopictus* borne diseases, and vector control remains the most efficient strategy for preventing arbovirus infections [4]. However, the long-term and heavy use of pesticides has led to severe resistance to commonly used larvicides and adulticides [4]. Furthermore, the population of *Ae*. *albopictus* is difficult to eradicate once established in an area [11]. Due to all these control challenges, novel strategies developed on increased knowledge of the biology, ecology, and vector competence are required.

Long noncoding RNAs (lncRNAs) are typically defined as a heterogeneous class of transcripts that are more than 200 nucleotides in length and lack the potential for protein coding [12–14]. According to their genomic locations with respect to their neighboring protein-coding genes, lncRNAs are generally classified into long intergenic ncRNAs (lincRNAs, transcripts mapped to the annotated intergenic regions), intronic lncRNAs (transcripts mapped completely within the introns of the known protein-coding genes), antisense lncRNAs (transcripts mapped to the exon of a protein-coding gene but on the opposite strand) and enhancer RNAs [13,15,16]. LncRNAs have been well characterized in many organisms and verified to be relatively less conserved across species [14,17–22]. LncRNAs can bind to DNA, RNA, and proteins to regulate gene expression at both the transcriptional and post-transcriptional levels [23]. Increasing evidences have demonstrated that lncRNAs play important roles in various biological processes and are essential for regulating gene expression [19]. It has been proven that lncRNAs not only function in the developmental processes of honey bees but also play important roles in regulating the development, behavior, stress resistance, sex identification, and dosage compensation of *Drosophila* [14,24,25]. Although there have been many studies on lncRNAs, a detailed characterization of lncRNAs in non-model organisms is somewhat limited [20], and the role of lncRNAs in most arthropods is still limited [14,26].

The identification of lncRNAs has been made possible in mosquitoes, studies in *Ae*. *aegypti* have demonstrated that lncRNAs are involved in early embryonic development and participate in virus-host interactions [20,27–29]. Regarding to *Ae*. *albopictus*, knowledge on lncRNAs is still limited. Xu et al. [30] reported specifically expressed lncRNAs in males and females. Azlan et al. [29] have identified lncRNAs of *Ae*. *albopictus* that participate in the regulation of genes involved in immunity and metabolic and cellular processes, affecting the infection and replication of DENV and ZIKV. However, knowledge regarding the genome-wide expression of lncRNAs in developmentally regulated *Ae*. *albopictus* remains unknown.

Due to the significant heterogeneity of lncRNAs, an in-depth understanding of the temporal expression profiles of lncRNAs throughout different time points is undoubtedly pivotal to predicting the mechanisms underlying developmental processes and can be used as targets to improve efficient and sustainable mosquito control strategies. Considering that some lncRNAs can be discovered only during narrow developmental time windows [31], samples of five developmental time points (including eggs, early larvae, late larvae, pupae, and adults) that cover all life cycles were used in this study to explore the lncRNA profiles and potential functions in *Ae*. *albopictus*.

## Materials and methods

### Sample preparation

The *Ae*. *albopictus* colony (Shandong, China) used in this study was reared at 27 ± 1°C and 65% relative humidity (RH) with a daily photoperiod of 14:10 h (L:D). The larvae were reared in dechlorinated tap water in plastic containers and fed a slurry that was mixed with of pork liver powder (homemade), yeast and distilled water. Pupae were collected individually, placed in plastic tubes filled with dechlorinated tap water and covered with absorbent cotton, until the adults emerged. Unfed adults (male and female) were collected two days after emergence. Adult females were supplied with blood by feeding on an anesthetized mouse, which was then returned to its cage. The experimental protocol for all animal experiments was in accordance with guidelines and with permission from the local animal ethics committee and with permission from the Institutional Animal Care and Use Committee of Shandong First Medical University. Eggs were collected within 24 h after damp collection filter paper was placed into an insect cage and were pooled to represent the embryonic stage. Larvae samples were divided into early (I–II instars) and late (III–IV instars) larval stages. Pupae samples were mixed pools of varied ages (1–3 days). Two hundred eggs, 20 early larvae, 20 late larvae, 10 pupae, 10 females and 10 males per pool, and three replicons per stage were prepared. All samples were flash frozen in liquid nitrogen immediately following collection and then stored at -80°C until RNA isolation.

### RNA extraction, library construction and sequencing

Total RNA was extracted separately from five groups of samples (eggs, early larvae, late larvae, pupae, and adults) using TRIzol Reagent (Invitrogen, CA, USA) according to the manufacturer’s protocol. RNA degradation and contamination were assessed using 1% agarose gels. RNA purity and concentration were then examined using an ND-1000 spectrophotometer (NanoDrop 2000, Thermo Fisher Scientific, MA, USA). RNA quantity was determined using the RNA Nano 6000 Assay Kit of the Bioanalyzer 2100 system (Agilent, CA, USA). RNAs of satisfactory quality were treated using the Ribo-Zero Gold Kit (Illumina, CA, USA) to eliminate rRNAs. Sequencing libraries were constructed with 1 μg of RNA using the NEBNext Ultra Directional RNA Library Prep Kit (NEB, Beijing, China) for Illumina. Sequencing was performed by Novogene (Beijing, China) using the Illumina HiSeq 2000 platform, and 150-bp paired-end (PE150) reads were obtained.

### Mapping and transcriptome assembly

The quality control of raw data was performed by removing low-quality reads, adaptor sequences, empty reads and rRNA reads using Trimmomatic v0.38 [32]. Clean reads were obtained by removal of reads containing adapter, poly-N and low quality reads from the raw data. Low quality reads (Phred score < 20; read length < 50 bases) were removed. All downstream analyses were based on clean data. The Q30 and GC contents of each sample were calculated. Clean reads were individually mapped to the *Ae*. *albopictus* genome (Genome version: AalbF2, assembly: GCA_006496715.1, NCBI) [33] using HISAT2 v2.1.0 [34]. The mapped reads were assembled by StringTie v1.3.3 [35]. Assembled transcripts were then merged into consensus transcripts using Cuffmerge [36]. Strict screening and filtering of the assembled transcripts were performed to accurately identify lncRNAs; for example, transcripts mapped within the 1 kb flanking regions of an annotated gene were removed. In particular, a minimum of 200 bp was set for the length of the assembled transcripts, and the CNCI [37], Pfam [38] and CPC [39] databases were used to exclude transcripts with protein-coding capability. The characteristics of novel lncRNAs were compared with known lncRNAs and mRNAs and named according to the rules of HGNC (The HUGO Gene Nomenclature Committee). Based on the results of sequence reads and alignments with the reference genome, together with the positional relationship of the lncRNA transcript to its neighboring genes, transcripts were classified as intergenic, intronic, and antisense lncRNAs.

### Identification of differentially expressed lncRNAs

The expression levels of transcripts were quantified by values of fragments per kilobase of transcript sequence per million base pairs sequenced (FPKM) values using StringTie v1.3.3. Differential expression analysis was performed using EdgeR [40]. *P*-values were adjusted using the Benjamini and Hochberg [41] procedure to control for false discovery. Differentially expressed (DE) lncRNAs were identified based on the threshold of |log2 (fold change) | > 0 and padj < 0.05 to include more lncRNAs. Venn diagrams were used to visualize the DE lncRNAs in difference comparisons. Hierarchical clustering heatmaps generated by R package version 1.0.8 software (https://cran.rproject.org/web/packages/pheatmap/) were used to display the DE lncRNAs.

### Co-expression analysis

Weighted gene co-expression network analysis (WGCNA) 1.4.7 was used to perform co-expression analysis of lncRNAs [42,43]. The gene expression profiles were imported into WGCNA to construct co-expression modules, with the threshold for the determination of weighted adjacency matrix fixed at soft power. The topological overlap matrix (TOM) was constructed by calculation based on the adjacency matrix. Then, gene modules were detected based on the TOM matrix, and the network connectivity of genes was calculated and divided into modules with similar expression patterns through hierarchical clustering analysis. Correlation analysis between each module and different time stages was also performed to explore modules that were highly related to developmental time points of *Ae*. *albopictus*. The association between module eigengenes (ME) and differentiation traits was measured according to Spearman’s correlation coefficient.

### Functional annotation and enrichment

To investigate the biological functions of different modules, Gene Ontology (GO) and Kyoto Encyclopedia of Genes and Genomes (KEGG) enrichment analyses were implemented by the clusterProfiler R package [44,45]. Target genes of lncRNAs involved in each module were used to conduct GO and KEGG analyses, and GO terms and KEGG pathways with a *p* value < 0.05 were considered significantly enriched.

### Construction of the interaction network and hub lncRNs identification

Gene connectivity of each module was represented by edge weight and defined as the sum of weights across all edges of a node in the gene co-expression network analysis. Hub lncRNAs were defined as lncRNAs with the highest intramodular connectivity in each functional module, which was calculated by the WGCNA algorithm identified by the Cytohubba plugin [46] in Cytoscape v3.7.2 [47]. The molecular complex detection (MCODE) plugin [48] in Cytoscape was applied to identify the ‘hub gene cluster’ which has a high degree of connectivity in a given network.

### Reverse transcription and real-time quantitative PCR (qRT–PCR)

Eight differentially expressed lncRNAs from RNA-seq analysis were randomly selected for qRT–PCR. The DE lncRNAs were validated using the same RNA samples used for Illumina sequencing. All experiments were performed in three biological and technical replicates. *β*-*Actin* was used as a reference gene, and all qRT–PCR runs were carried out on an ABI7500 qRT–PCR platform. The 2^−ΔΔCT^ method was used to estimate the relative expression of each lncRNA [49].

## Results

### Genome-wide identification of lncRNAs

To systematically explore the expression profile of lncRNAs throughout different developmental stages, a total of 18 libraries from five time points (with three biological replicates for each time point) were constructed. Illumina sequencing yielded a total of 1,722,204,342 raw reads and 258.32 Gb of raw data. Raw RNA-seq sequences were deposited in the NCBI Sequence Read Archive (SRA) database with accession number PRJNA757239. There were 1,697,076,094 clean reads and 254.57 Gb of clean data were obtained after quality trimming and filtering. The Q30 of all samples was above 90.98%, and 42.17% to 67.01% of reads of different samples were mapped to the genome of *Ae*. *albopictus* (S1 Table).

Altogether, 21,414 putative lncRNAs and 45,234 mRNAs were obtained and used for subsequent analysis. After filtering out the known lncRNAs and mRNAs, 13,805 novel lncRNAs and 4,431 mRNAs were identified (S2 Table). These novel lncRNAs and mRNAs may be valuable for understanding the development of *Ae*. *albopictus*.

### Characteristics of the identified lncRNAs

**Fig 1:**
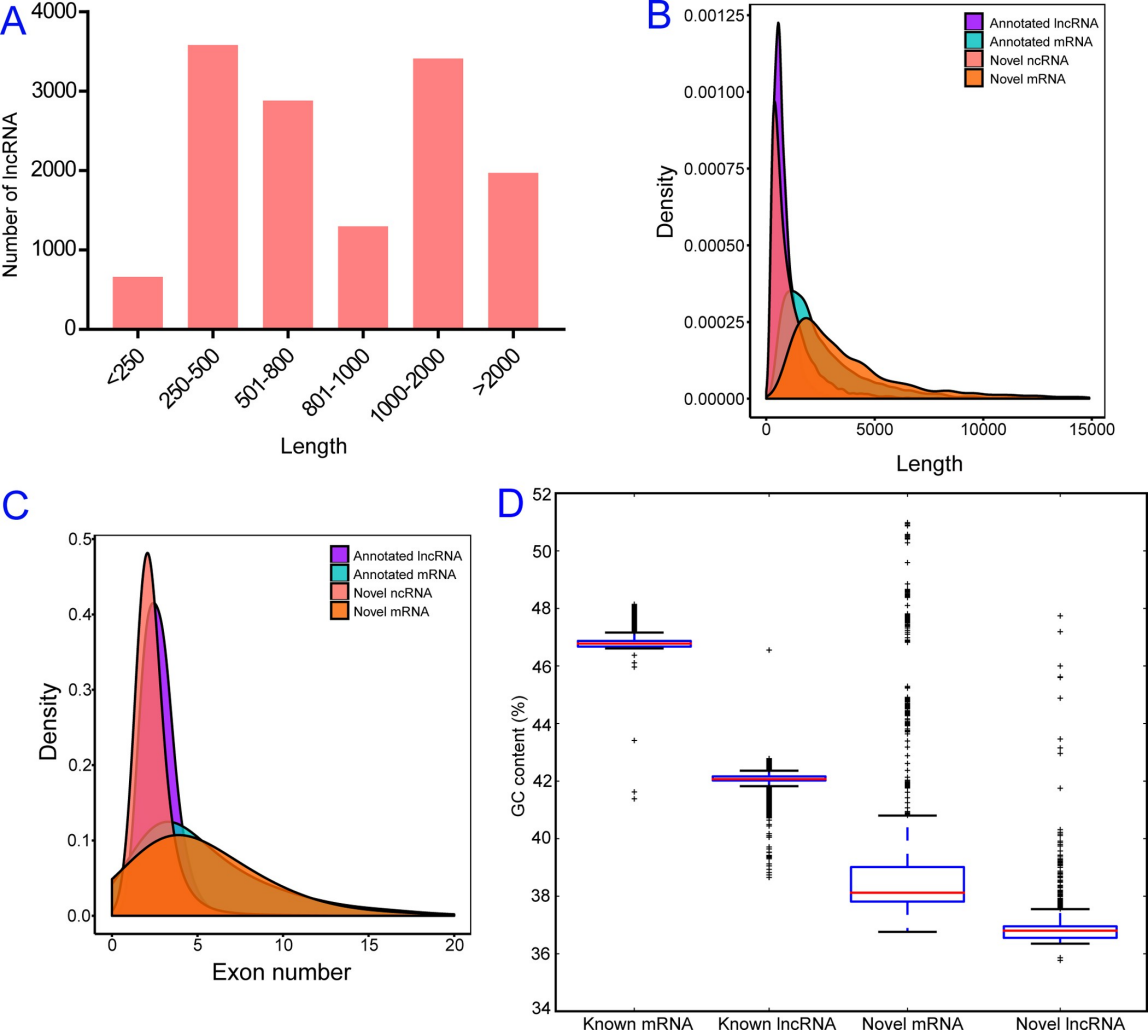
Features of *Aedes albopictus* lncRNAs. (A) Transcript length distribution of putative lncRNAs; (B) Distribution of transcript length of lncRNAs and mRNAs to verify the length of novel lncRNAs and mRNAs are confirmed with that annotated; (C) Density plot of exons distribution of lncRNAs and mRNAs to verify the length of novel lncRNAs and mRNAs are confirmed with that annotated; (D) Mean GC contents of putative lncRNAs and mRNAs. Error bars represent 95% confidence intervals.

According to their genomic location, all novel lncRNAs were cataloged as lincRNAs (9,132, 66.15%), sense overlapping (3,214, 23.28%), and antisense (1,459, 10.57%) (S1 Fig). The investigation of the genome distribution of lncRNAs demonstrated that 21,414 putative lncRNAs were assembled on 1,058 transcripts of the genome of *Ae*. *albopictus* (S3 Table). The length of lncRNAs ranged from 201 bp to 30,487 bp, and the length of most of the lncRNAs was shorter than 800 bp (Fig 1A). The mean length of lncRNAs was 1,167 bp, approximately a quarter of the length of mRNAs (4,843 bp) (Fig 1B). In addition, lncRNAs had fewer exons and lower GC contents than mRNAs (Fig 1C and 1D). Analysis of their distribution in the genome suggested that most lncRNAs were located on the largest scaffold, NW_021837045.1 (1,475 lncRNAs), followed by NW_021838153.1 (1,234 lncRNAs) and NW_021838576.1 (1,040 lncRNAs) (S2 Fig and S2 Table).

### Expression profile of the lncRNAs by developmental stage

**Fig 2:**
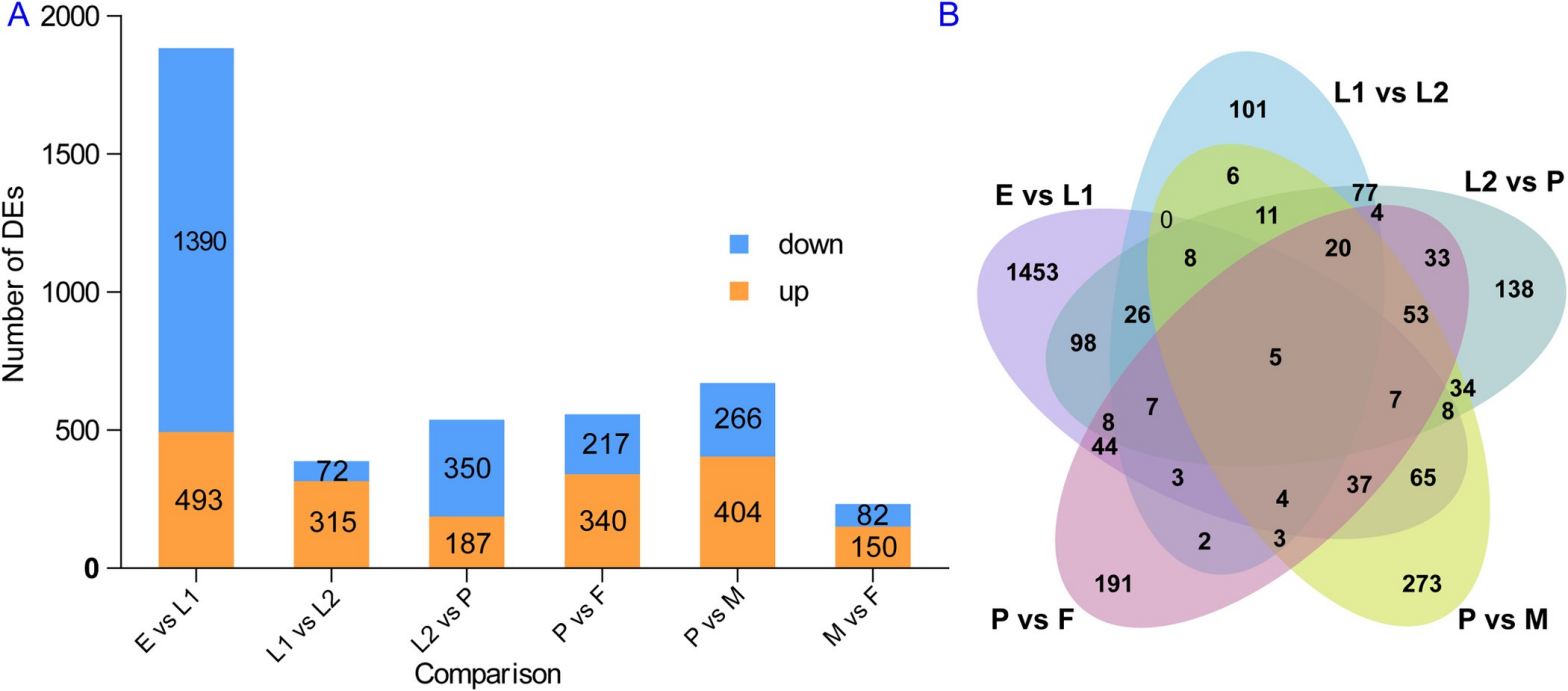
Expression profiles of *Aedes albopictus* putative lncRNAs. (A) Histogram showing DE lncRNAs among successive developmental time points; (B) Venn diagram displaying the number of overlapping DE lncRNAs between different groups. E, egg; L1, early larvae; L2, late larvae; P, pupae; F, female; M, male.

In total, 4,034 DE lncRNAs were found: 1,883 between eggs and early larvae, 387 between early and late larvae, 537 between late larvae and pupae, 557 between pupae and females, 670 between pupae and males, and 232 between females and males (Fig 2A). Both the most up- and down-regulated lncRNAs were found between eggs and early larvae (493 up- and 1,390 down-regulated). The fewest of up-regulated lncRNAs were found between females and males (150 up- and 82 down-regulated), and early and late larvae showed the fewest of the down-regulated DE lncRNAs (315 up- and 72 down-regulated) (Fig 2A). DE lncRNAs between any two successive developmental time points were mostly unique compared to other pairwise time point comparisons (Fig 2B).

Given the expression characteristics of lncRNAs, co-location and co-expression target genes of DE lncRNAs were selected firstly. Altogether, 18,993 lncRNAs and 82,474 mRNAs were filtered according to whether the lncRNAs could regulate the expression of overlapping or nearby mRNAs (within 100 kb). Then, the co-expression correlation between lncRNAs and mRNAs was evaluated using Pearson correlation (>0.95), and 5,922 lncRNAs co-expressed with 11,895 target mRNAs (S4 Table).

Hierarchical clustering results of DE lncRNAs and their target mRNAs showed significant developmental stage-specific characteristics (S3A Fig). There were obvious differences in lncRNAs expressed in eggs at other stages, indicating that lncRNAs were specific to this stage. Some lncRNAs were co-expressed in early and late larvae, while both larval stages had specific lncRNAs (S3A Fig). Variable expression levels of lncRNAs at the pupal, female and male developmental stages were also observed in the expression heatmap (S3A Fig). In comparison, the expression profile of lncRNAs was not as markedly varied as that of mRNAs, which exhibited distinct temporal specificity (S3B Fig).

### Correlation of differentially expressed lncRNAs and biological function annotation

**Fig 3:**
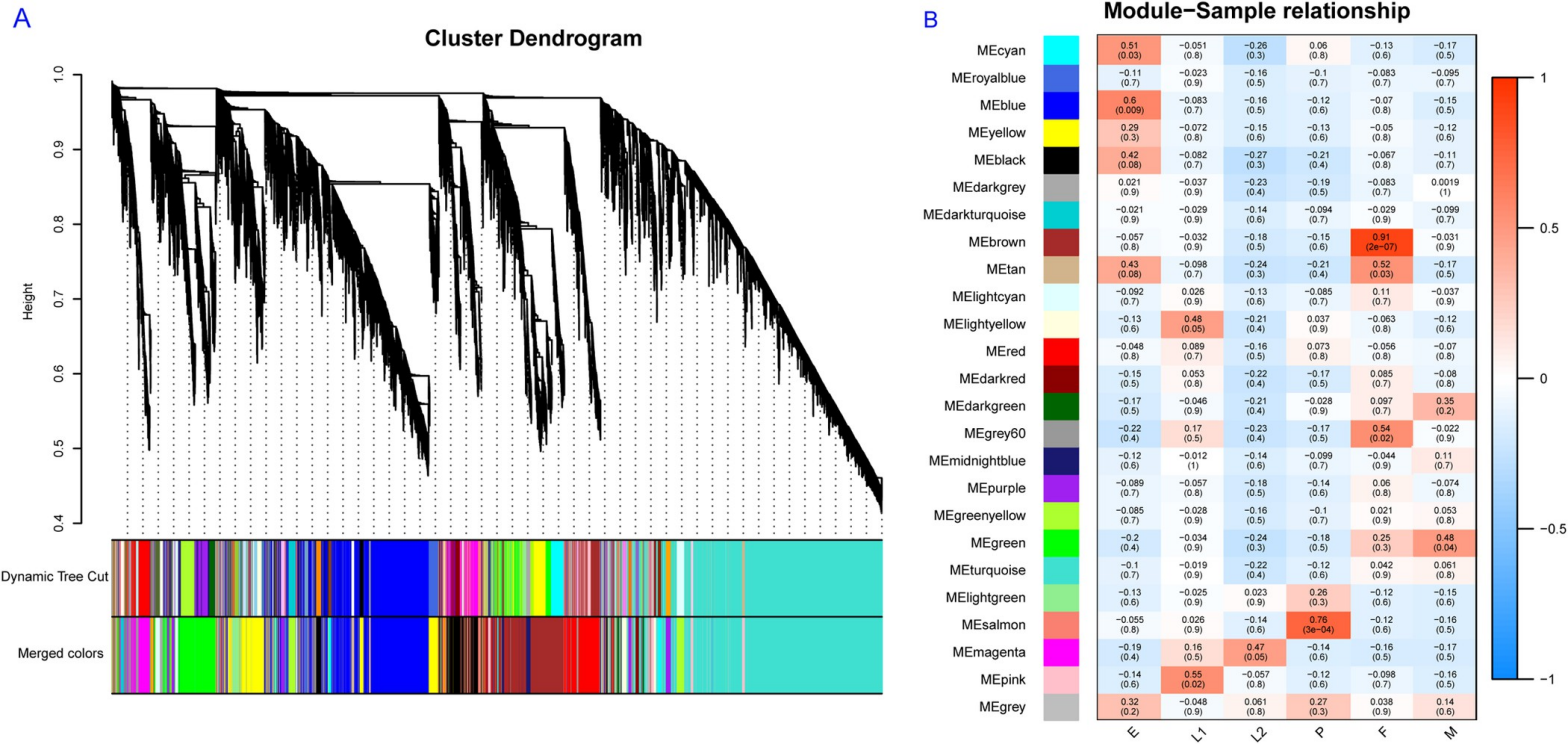
Gene modules identified by WGCNA. (A) Dendrogram of putative lncRNAs cluster obtained by hierarchical clustering, 25 modules were identified by different colors; (B) Module-trait relationships and corresponding *p* values. Each module was represented by one color on the left site of the heatmap. The color scale on the right shows module–trait correlation. Red indicated positive correlation (correlation > 0), whereas blue color indicated negative correlation (correlation < 0). E, egg; L1, early larvae; L2, late larvae; P, pupae; F, female; M, male.

A broad time series of sampling made it possible to follow the expression dynamics of lncRNAs as development proceeded. WGCNA was used to explore highly associated modules and predict the potential roles of lncRNAs in the development of *Ae*. *albopictus*. According to the scale-free topology criteria [50], soft power 5 (scale-free *R*^*2*^ > 0.85) was chosen as the soft threshold to establish a weighted adjacency matrix. Overall, 25 modules were obtained, the module size ranged from 26 to 1893, and the unclustered lncRNAs were grouped into grey module (Fig 3A and S5 Table). The expression profile of different modules further demonstrated the correlation between lncRNAs and developmental time points. According to the results of Spearman’s correlation analysis, 6 modules exhibited statistically significant correlations (*P*< 0.05) with specific developmental time points (Fig 3B). The modules that had the highest correlation coefficient were selected for further research, including blue, pink, magenta, salmon, brown, and green module, which correlated to eggs (E), early larvae (L1), late larvae (L2), pupae (P), females (F), and males (M), respectively.

To obtain insight into the biological function of these modules, GO term enrichment and KEGG pathway analyses of lncRNAs were performed. The blue module was highly associated with eggs. Functional enrichment analysis suggested that significantly enriched GO terms were belong to CC category, such as nucleus, membrane-bounded organelle and intracellular membrane-bounded organelle (Table 1 and S4A Fig). Consistently, KEGG pathway enrichment analysis revealed highly enriched pathways in the blue module, including spliceosome, Wnt signaling pathway, and base excision repair (Table 1 and S4B Fig).

**Table 1:** Top 5 GO terms and KEGG pathways of the blue, pink, magenta, salmon, brown and green module.

Module	GO term			KEGG pathway
	GO accession	Category	Description	
blue	GO:0005634	CC	nucleus	Spliceosome
	GO:0043227	CC	membrane-bounded organelle	Wnt signaling pathway
	GO:0043231	CC	intracellular membrane-bounded organelle	Base excision repair
	GO:0043229	CC	intracellular organelle	Pyrimidine metabolism
	GO:0043226	CC	organelle	RNA transport
pink	GO:0042302	MF	structural constituent of cuticle	Valine, leucine and isoleucine degradation
	GO:0005198	MF	structural molecule activity	Fatty acid metabolism
	GO:0008061	MF	chitin binding	Propanoate metabolism
	GO:0006030	BP	chitin metabolic process	Citrate cycle (TCA cycle)
	GO:1901071	BP	glucosamine-containing compound metabolic process	Pyruvate metabolism
magenta	GO:0003824	MF	catalytic activity	Valine, leucine and isoleucine degradation
	GO:0006030	BP	chitin metabolic process	Propanoate metabolism
	GO:1901071	BP	glucosamine-containing compound metabolic process	Fatty acid degradation
	GO:0006040	BP	amino sugar metabolic process	Glycine, serine and threonine metabolism
	GO:0008061	MF	chitin binding	Metabolism of xenobiotics by cytochrome P450
salmon	GO:0004252	MF	serine-type endopeptidase activity	Sphingolipid metabolism
	GO:0006040	BP	amino sugar metabolic process	Lysosome
	GO:0006030	BP	chitin metabolic process	Fatty acid biosynthesis
	GO:1901071	BP	glucosamine-containing compound metabolic process	Glycosphingolipid biosynthesis—globo series
	GO:0008236	MF	serine-type peptidase activity	Synthesis and degradation of ketone bodies
brown	GO:0005488	MF	binding	Dorso-ventral axis formation
	GO:0003676	MF	nucleic acid binding	Fanconi anemia pathway
	GO:0000723	BP	telomere maintenance	Neuroactive ligand-receptor interaction
	GO:0032200	BP	telomere organization	Tyrosine metabolism
	GO:0060249	BP	anatomical structure homeostasis	Nitrogen metabolism
green	GO:0016021	CC	integral component of membrane	Ribosome
	GO:0031224	CC	intrinsic component of membrane	Neuroactive ligand-receptor interaction
	GO:0016020	CC	membrane	Phototransduction—fly
	GO:0044425	CC	membrane part	Circadian rhythm—fly
	GO:0004930	MF	G-protein coupled receptor activity	Purine metabolism

The pink module was mainly related to early larvae. In the GO term enrichment analysis, more lncRNAs were assigned to the MF and BP categories in this module, and fewer terms were enriched in the CC category (S4A Fig). LncRNAs of this module were mainly enriched in structural constituent of cuticle and chitin metabolic process (Table 1). The KEGG pathway enrichment analysis indicated that most pathways in the pink module were involved in metabolism, such as valine, leucine and isoleucine degradation, fatty acid metabolism, propanoate metabolism, citrate cycle (TCA cycle), pyruvate metabolism, and extracellular matrix (ECM)-receptor interaction (Table 1 and S4B Fig). Similar to the pink module, the enriched GO terms in the magenta module, which were highly related to late larvae, were assigned to the BP and MF categories (Table 1 and S4A Fig). The highly enriched GO terms in this module included catalytic activity, chitin metabolic process and chitin binding (Table 1). In KEGG pathway analysis, the magenta module was mainly involved in metabolism-related pathways, such as valine, leucine and isoleucine degradation, propanoate metabolism and glycine, serine and threonine metabolism (Table 1 and S4B Fig).

The salmon module showed a high association with pupal stage. The enriched GO terms under the BP category were metabolism related, such as amino sugar metabolic process and chitin metabolic process. Serine-related (serine-type endopeptidase activity, serine-type peptidase activity) GO terms were advantageous under the MF category (Table 1 and S4A Fig). None of the terms under the CC category were significantly enriched. Most of the enriched KEGG pathways in the salmon module were metabolism-related, while the presence of pathways, such as lysosome and insect hormone biosynthesis, made this module differ from the abovementioned modules (Table 1 and S4B Fig).

With respect to the brown module, which was closely related to females, the highly enriched GO terms included binding, nucleic acid binding and telomere maintenance (Table 1 and S4A Fig). Dorso-ventral axis formation, neuroactive ligand-receptor interaction, lysosome, spliceosome, and homologous recombination were the most enriched KEGG pathways (Table 1 and S4B Fig).

The green module was mainly associated with males, and functional enrichment suggested that the top GO terms were membrane-related, including integral component of membrane, intrinsic component of membrane and membrance (Table 1 and S4A Fig). KEGG analysis showed that ribosome and neuroactive ligand-receptor interaction, phototransduction-fly, circadian rhythm-fly, and ECM-receptor interaction were the most enriched pathways in this module (Table 1 and S4B Fig).

Subsequently, the top 10 GO terms (BP, CC, and MF categories were ranked separately) and KEGG pathways were further compared to explore the same set of functions enriched in different modules. The results demonstrated that the pink (early larval stage) and magenta (late larval stage) modules shared the most enriched biological functions (14 GO terms and 4 KEGG pathways) (S5A and S5B Fig), followed by the blue (eggs) and brown modules (females) (8 GO terms and 3 KEGG pathways) (S5C and S5D Fig). Among the 14 GO terms enriched in both the pink and magenta modules, 10 GO terms were also enriched in salmon (pupal stage) (S5A and S5B Fig). In addition to metabolism-related GO terms, peptidase activity (GO:0008233), peptidase activity, acting on L-amino acid peptides (GO:0070011), chitin binding (GO:0008061), serine-type peptidase activity (GO:0008236), and serine hydrolase activity (GO:0017171) were involved in the development of all three modules. The enriched KEGG pathways in both the pink and magenta modules included pyruvate metabolism, fatty acid metabolism, propanoate metabolism and valine, leucine and isoleucine degradation.

All of the GO terms involved in the blue and brown modules were under the MF category (GO:0046914, transition metal ion binding; GO:0097159, organic cyclic compound binding; GO:1901363, heterocyclic compound binding; GO:0043169, cation binding; GO:0008270, zinc ion binding; GO:0046872, metal ion binding; GO:0003676, nucleic acid binding; and GO:0005488, binding). Homologous recombination (map03440) and spliceosome (map03040) were two KEGG pathways enriched in both the blue and brown modules. The enriched functions of the green module (males) were significantly different from those of the other stages, and only one KEGG pathway (map04080: Neuroactive ligand-receptor interaction) was co-expressed in the brown and green modules (S5C and S5D Fig).

### Network analysis and hub genes identification

**Fig 4:**
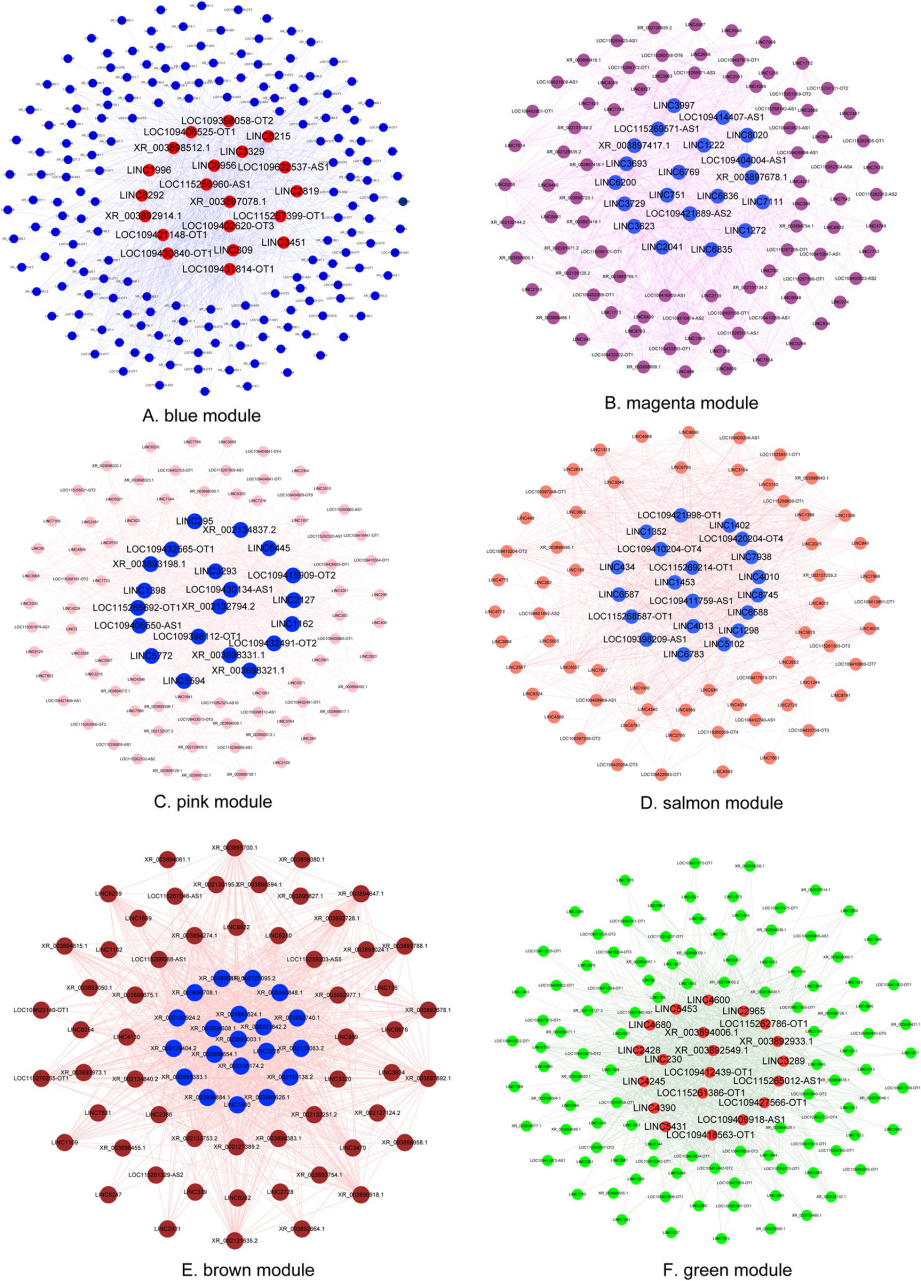
Co-expression network of blue module (A), magenta module (B), pink module (C), salmon module (D), brown module (D) and green module (F). Each node represents a lncRNA, each edge denotes a target relationship between lncRNAs. The top 20 hub genes were showed in red (in blue and green module) and blue (magenta, pink, salmon, and brown module).

Networks were constructed to explore the interaction of lncRNAs in each module. LncRNAs are represented with nodes, and the edges between genes represent co-expression correlations. The number of lncRNAs and edge weight were quite different among modules. According to the rank of weights, the top 1,000 nodes in each module were selected for network analysis. Hub genes were defined as lncRNAs that possessed high connectivity in each module (Fig 4). According to the rank of genes in the Cytohubba plugin, the top 20 hub lncRNAs in each module were listed in S6 Table. The lncRNAs with the highest intramodular connectivity in blue, pink, magenta, salmon, brown, and green module were LOC109406525-OT1, XR_002132794.2, LINC1272, LINC1298, XR_002132924.2 and LOC109412439-OT1, respectively.

Hub gene clusters within each network were identified using MCODE. The number of significant hub gene clusters was different among modules (S6 Fig). Four clusters were found in the blue module, followed by three modules in the magenta and salmon modules and two clusters in the pink module. There was only one cluster in the brown and green modules. The hub gene cluster of the brown module, which consisted of 41 lncRNAs, showed the highest score (36.8). The lowest score (3.2) was detected in the cluster 4 of the blue module, with six lncRNAs involved (S6 Fig).

### Validation of the gene expression results by qRT-PCR

**Fig 5:**
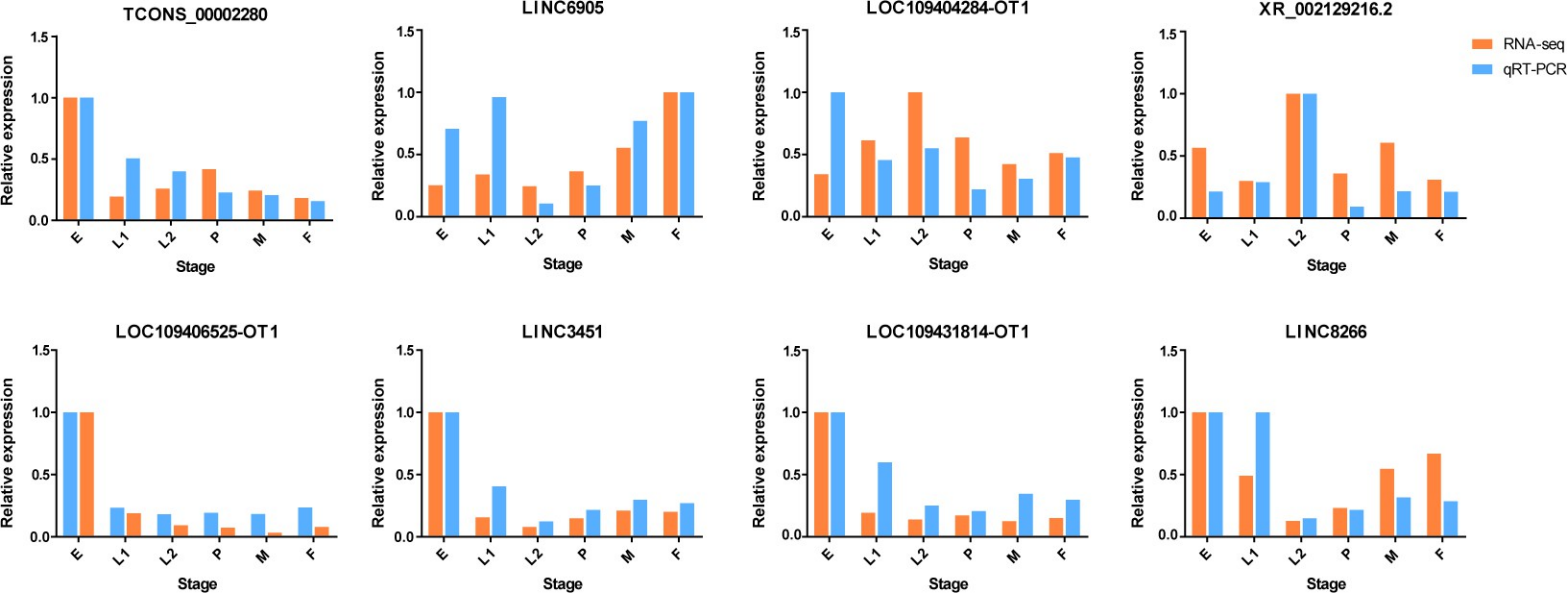
Comparison of expression profiles of eight genes by RNA-Seq and qRT-PCR analyses. Expression levels in eggs were adjusted to 1. The X-axis represented developmental stages, the Y-axis indicated the relative expression of each gene; orange bars were log2 (FPKM+1) values of RNA-Seq and the blue bars were relative expression of qPCR. E, egg; L1, early larvae; L2, late larvae; P, pupae; F, female; M, male.

To validate the sequencing data, 8 lncRNAs were randomly selected to examine the expression patterns at each developmental time point. A list of primers used in this study is shown in S7 Table. The relative expression of lncRNAs detected by qRT–PCR was compared with the expression profiles of RNA-seq. The qRT–PCR results demonstrated that the expression pattern of the 8 randomly selected lncRNAs was similar to that observed using the RNA-seq data (Fig 5), illustrating the reliability of the RNA-Seq data and guaranteeing the accuracy of the related analysis.

## Discussion

In the present study, we systematically investigated the lncRNA expression profile by RNA-seq spanning five developmental time points (eggs, early larvae, late larvae, pupae, and adults), providing a comprehensive picture of lncRNAs in *Ae*. *albopictu*s. Altogether, 21,414 putative lncRNAs were identified, among which 13,805 have not been previously reported. Many novel lncRNAs (66.1%) are located in intergenic regions. The lncRNAs of *Ae*. *albopictus* shared similar characteristics with lncRNAs in other species (Fig 1), including being short in length, having a lower GC content, being less conserved and being expressed at lower levels than mRNAs [17–21].

The expression profile of lncRNAs exhibited high stage- and sex-specific characteristics. Each time point showed a distinct lncRNA expression pattern; for example, there was a subset of lncRNAs that were highly enriched in eggs compared with all other stages (S3A Fig). In addition, many lncRNAs were prominently up-regulated at the onset of metamorphosis developmental stages (Fig 2A). Investigation of the expression profile across different time points revealed that fewer DE lncRNAs were identified between the same life stage, i.e., early and late larval stage, and adult sex (female and male). However, in accordance with the developmental phase changes, many more DE lncRNAs were observed between different stages, such as eggs vs. early larvae (493 up- and 1390 down-regulated), late larvae vs. pupae (187 up- and 350 down-regulated), pupae vs. females (340 up- and 217 down-regulated), and pupae vs. males (404 up- and 266 down-regulated) (Fig 2A). These differences may be caused by physiological changes during metamorphic development, and these DE lncRNAs may be crucial for the transition between stages. Similar developmental stage-related expression patterns have also been observed in *Ae*. *aegypti* [20] and *Anopheles gambiae* [51].

Co-expression analysis by WGCNA revealed that the expression patterns of lncRNAs were closely correlated with specific developmental time points, highlighting the significant developmental specificity of lncRNA expression (Fig 3B). Enrichment analysis for each module showed that several GO terms and KEGG pathways were enriched in both the blue and brown modules, which were mainly associated with eggs and females, respectively (S4A and S4B Fig). All of the co-expressed GO terms were under MF category related to binding functions, including but not limited to zinc ion binding, organic cyclic compound binding, and nucleic acid binding. Together with the enriched KEGG pathways, homologous recombination and spliceosome, suggesting that some lncRNAs may be maternally inherited and play critical roles in the duplication, transcription, and cell differentiation occurring during embryonic development [52]. Similar evidence of maternal inheritance of lncRNAs was also found in *Ae*. *aegypti* [20]. Functional annotation of the pink, magenta and salmon modules indicated that enriched GO terms and KEGG pathways were significantly associated with metabolism, coinciding with rapid metamorphic development during these stages. For instance, the enrichment of chitin metabolic processes and chitin binding are closely related to the formation and degradation of chitin, which is a major structural component of the insect cuticle that protects insects from chemical erosion, physical abrasion and pathogenic invasion [53,54] and serves as an attachment matrix for other cuticular proteins, plays an important role in molting during the larval-larval and larval-pupal stages [55,56].

In addition to the co-expressed functions with other modules, the enriched GO terms and KEGG pathways in the blue module indicated intense cell differentiation in the egg stage (S4 Fig), which was potentially responsible for organogenesis during the embryonic stage. We further confirmed that lncRNAs are involved in developmental regulation [14,17,20]. The enriched functions in the pink module that were mainly associated with the early larval stage exhibited potential roles in diverse processes, such as the response to biotic stimulus, and defense response to other organism were immune-related. The emergence of immune response may contribute to protect cell or organism from damage caused by other organisms. Moreover, the enriched structural constituent of cuticle, meiotic chromosome segregation extracellular matrix, and highly associated ECM-receptor interaction and proteinaceous extracellular matrix pathways indicated the involvement of tissue and organ morphogenesis at the early larval stage. Comparison between the pink and magenta modules revealed that the biological functions of the late larvae were mainly focused on metabolism-related processes, such as catalytic activity and protein metabolic process (S4A Fig), which are necessary for development. Therefore, we speculate that part of the organogenesis and formation of regulation networks may have occurred in early larvae (I and II instars), whereas individual growth was mostly took place in the later larval stage (III and IV instars).

The salmon module was highly associated with pupal stage. Except for functions co-expressed with larval stages, metabolism-related GO terms were dominant in this module (S4A Fig), which may have contributed to pupal growth, similar to that in the magenta module. The enriched pathways of sphingolipid metabolism and synthesis and degradation of ketone bodies are involved in lipid metabolism, and sphingolipids are intimately tied to intracellular membrane transport and signaling [57]. Glycosphingolipids (glycosphingolipid biosynthesis pathway) are key components of eukaryotic cellular membranes [58], while the fatty acid biosynthesis pathway is an essential cellular process that converts nutrients into metabolic intermediates for membrane biosynthesis, energy storage and the generation of signaling molecules [59]. Therefore, we deduced that lncRNAs in this module participate in the preparation of metamorphic development from pupae to adults, consisting with the hypothesis that lncRNAs are involved in regulating the timing of developmental transition [17]. Additionally, the enriched lysosome pathway suggested a fully activated immune response.

Functional annotation demonstrated that lncRNAs of the green module, which was highly related to males, were significantly different from all other modules. The neuroactive ligand-receptor interaction pathway was co-expressed in both the green and brown modules, indicating similar neuro function between males and females [60]. Additionally, the phototransduction-fly and circadian rhythm-fly pathways that are essential for the survival of adult mosquitoes were highly enriched in this module (S4B Fig).

LncRNAs have emerged as important regulators of gene expression and have proven to be a key to unlocking many underlying molecular mechanisms of development [19,61–64]. However, due to the versatile nature of regulation, which adds to the complexity of understanding lncRNAs, only a few lncRNAs have been thoroughly mechanistically characterized to date, with even fewer functionally verified lncRNAs. The contribution of lncRNAs to development renders them both intriguing and challenging to characterize [65–67]. This study provides the most comprehensive catalog of candidate lncRNAs and their expression patterns and potential functions across multiple life stages and both sexes of *Ae*. *albopictus*. Expression profiling showed that lncRNAs were expressed in a much narrower time period, as lncRNAs demonstrated specific expression characteristics following the developmental process, and six modules that were significantly correlated with different time points were identified. Predicted biological functions, hub lncRNAs and hub gene clusters of each module will spur further investigations on the role of lncRNAs during the metamorphosis of *Ae*. *albopictus*.

In conclusion, the results of this study expand our understanding of lncRNAs in *Ae*. *albopictus* and provide the basis for future studies of noncoding RNAs. The functional enrichment of lncRNAs will provide theoretical support for detailed studies on how lncRNAs are involved in the regulation of the developmental transition of *Ae*. *albopictus*. The hub lncRNAs and hub gene clusters identified as corresponding to six modules are supposed to play crucial roles at different developmental time points, which can be used as time-specific tuners by governing the timing of developmental transitions, and this knowledge will contribute to improving mosquito control strategies [14]. However, little is currently known regarding the function of lncRNAs and their role in the development of mosquitoes. The effective use of hub lncRNAs as targets for mosquito control requires a considerable amount of research to elucidate their regulatory mechanism in the metamorphosis of *Ae*. *albopictus*. Perturbation of the expression of lncRNAs, either by overexpression or knockdown might leads to gain or loss of functions, and qRT-PCR or deep sequencing are needed to observe changes in gene expression and potential biological functions of lncRNAs. In addition, many lncRNAs have also been reported to be tissue specific, while the currently used method usually considers all lncRNA expression. Therefore, sequencing technology at the single-cell level would provide more information regarding the dynamic changes in lncRNAs.

With a growing number of lncRNAs functionally characterized in humans, several lncRNAs have been used as therapeutic targets and biomarkers for cancer therapy [68–71]. With the unveiling of functions and mechanisms, lncRNAs can be explored as a new alternative for the population control of mosquitoes.

## Supporting information

S1 Fig Classification of lncRNAs based on their location on the genome of *Aedes albopictus*. (TIF)

S2 Fig The sum of putative lncRNAs distribution on different *Aedes albopictus* genome scaffolds. (TIF)

S3 Fig The expression pattern of lncRNAs (a) and mRNAs (b) in different developmental stages and displaying in hierarchical clustering. Each row represents one lncRNA and each column represents one sample; −4, −2, 0, 2, and 4 represent fold change. Red indicates high expression and blue represents low expression. E1-E3, egg; L1-1, -2, -3, early larvae; L2-1, -2, -3, late larvae; P1-P3, pupae; F1-F3, female; M1-M3, male.
(TIF)

S4 Fig Enrichment analysis of the blue, brown, pink, magenta, salmon and green module. (a) Top 10 GO terms under BP (blue), CC (red) and MF (green) categories; (b) Top 10 KEGG pathways. The different colors from blue to red represent the Q value (false discovery rate value). The different sizes of the round shapes represent the number of genes in a pathway.
(TIF)

S5 Fig Venn diagram showing the enriched GO terms and KEGG pathways in six modules highly correlated with different developmental stages (in bracket). (a) co-expressed GO terms in the magenta, pink and salmon module; (b) co-expressed KEGG pathways in the magenta, pink and salmon module; (c) co-expressed GO terms in the brown, blue and green module; (d) co-expressed KEGG pathways in the brown, blue and green module.
(TIF)

S6 Fig Hub gene clusters of modules extracted by MCODE. Blue module: a1-a4; magenta module: b1-b3; pink module: c1-c2; salmon module: d1-d2; brown module: e1; green module: f1. Each node represents a lncRNA, each edge denotes a target relationship between lncRNAs.
(TIF)

S1 Table Data statistics of samples for RNA sequencing. (XLS)

S2 Table List of novel and known lncRNAs and mRANs. (XLS)

S3 Table Number of lncRNA transcripts distributed on genome of *Aedes albopictus*. (XLSX)

S4 Table Co-expressing DE lncRNAs and target mRNAs. (XLS)

S5 Table Number of lncRNAs of each module. (XLS)

S6 Table Top 20 hub lncRNAs in different modules. (XLS)

S7 Table Primers used for qRT-PCR in this study. (XLS)
