# Supplementary figures and images for: Systematic identification and characterization of long noncoding RNAs (lncRNAs) during Aedes albopictus development
Source: PLoS Negl Trop Dis. 2022 Apr 13;16(4):e0010245. doi: 10.1371/journal.pntd.0010245 (PMC9007367; doi:10.1371/journal.pntd.0010245)

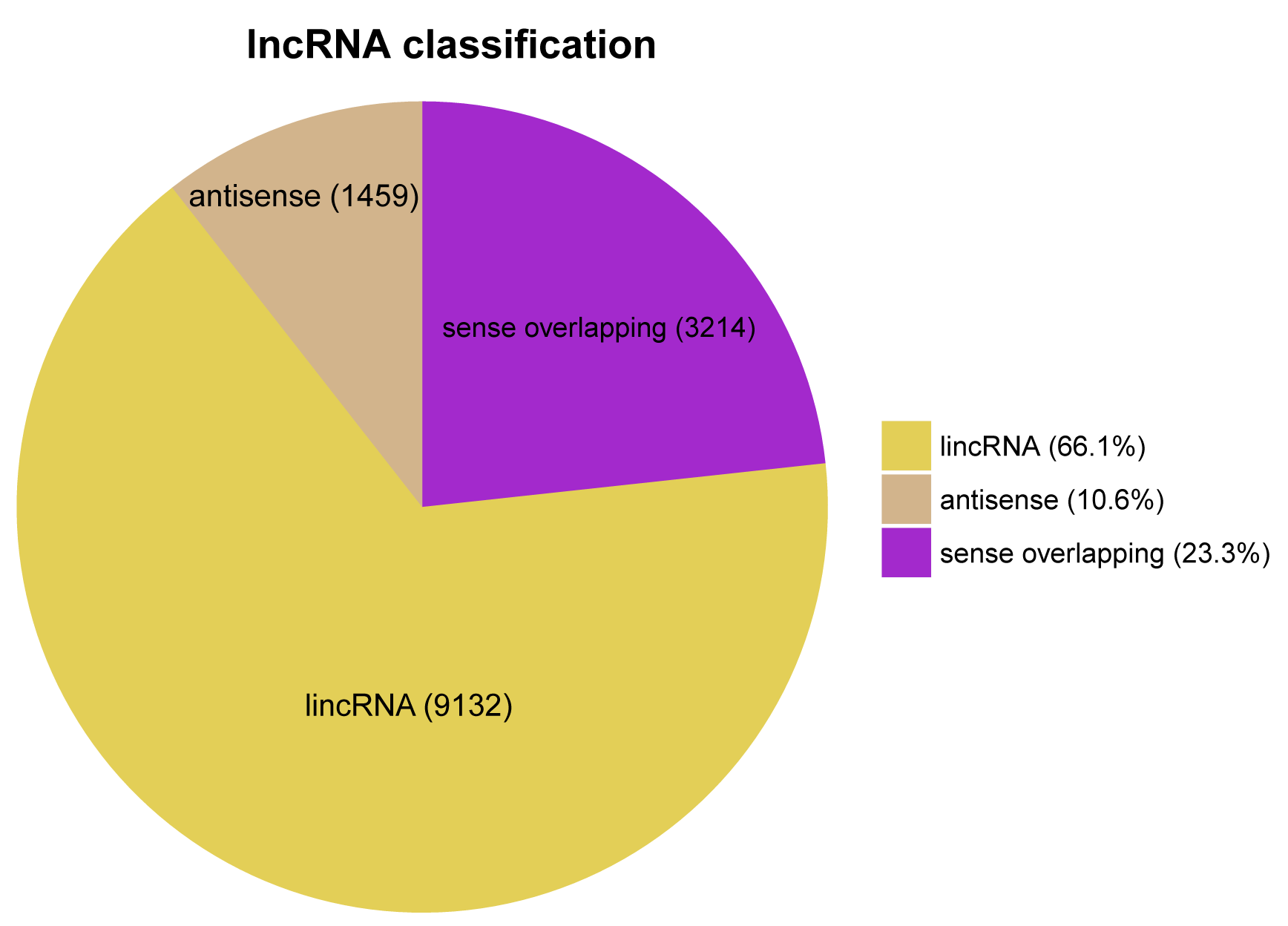

Supplement: S1 Fig — (TIF) [file pntd.0010245.s001.tif]

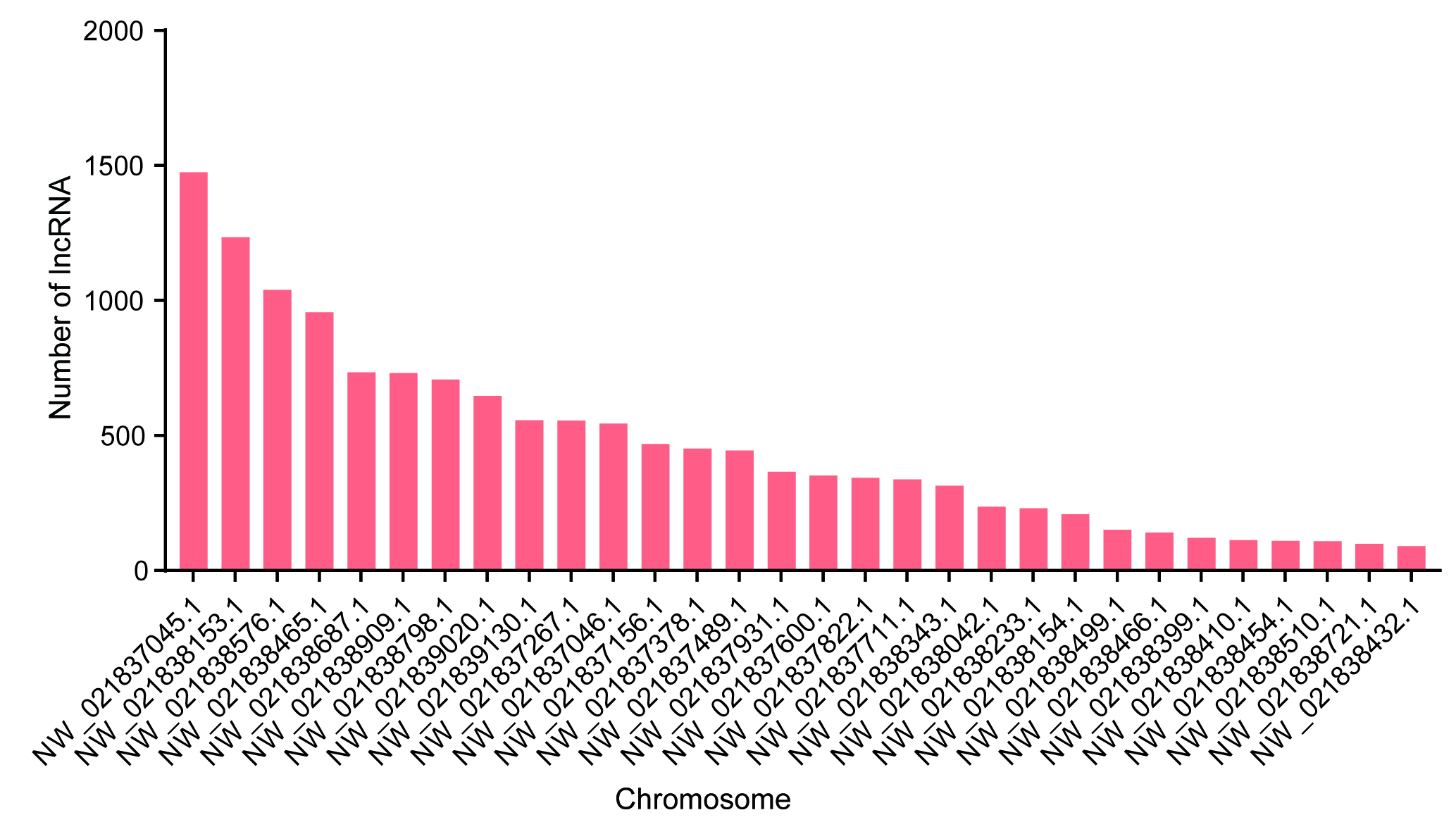

Supplement: S2 Fig — (TIF) [file pntd.0010245.s002.tif]

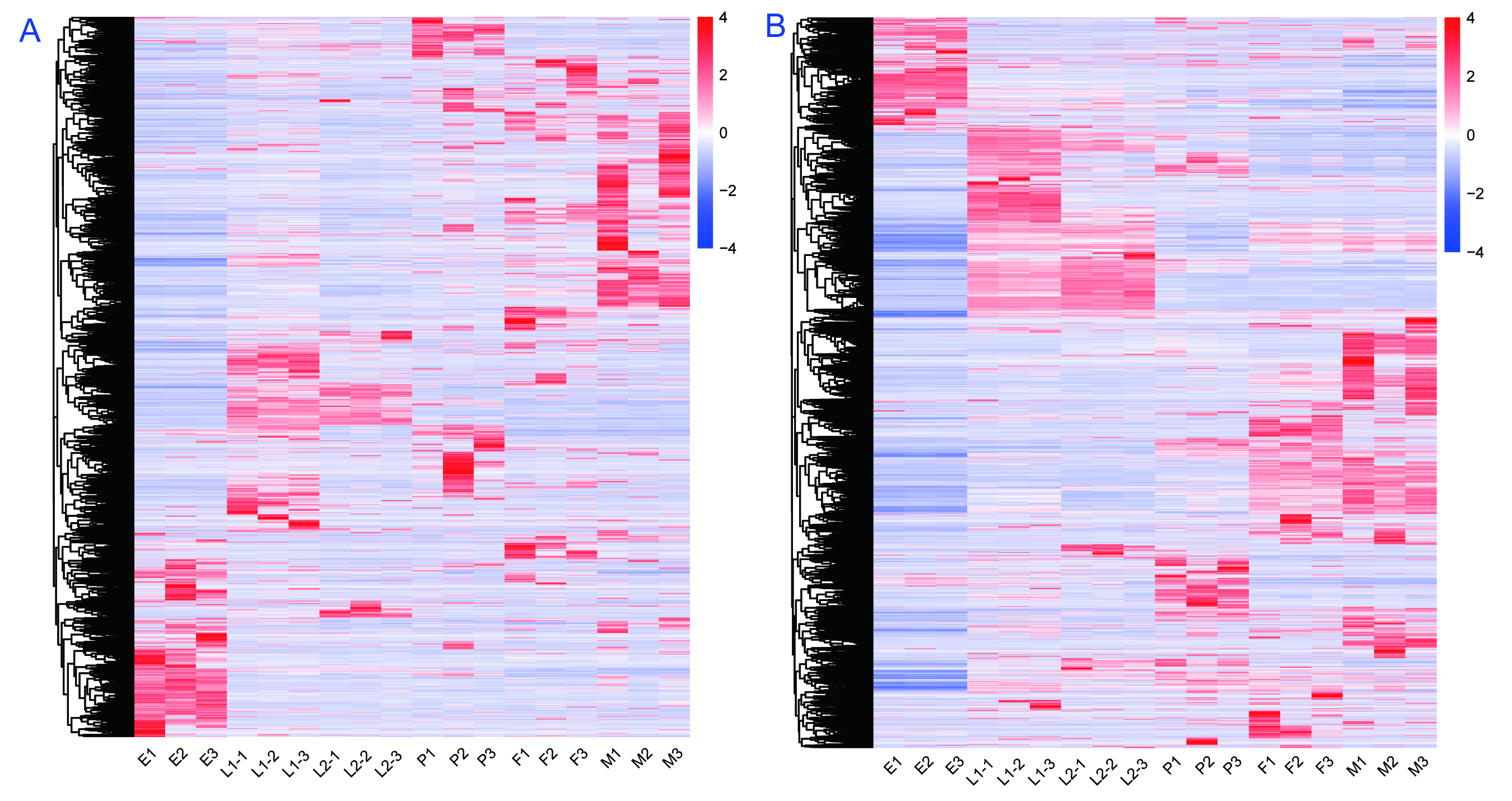

Supplement: S3 Fig — The expression pattern of lncRNAs (a) and mRNAs (b) in different developmental stages and displaying in hierarchical clustering. Each row represents one lncRNA and each column represents one sample; −4, −2, 0, 2, and 4 represent fold change. Red indicates high expression and blue represents low expression. E1-E3, egg; L1-1, -2, -3, early larvae; L2-1, -2, -3, late larvae; P1-P3, pupae; F1-F3, female; M1-M3, male. (TIF) [file pntd.0010245.s003.tif]

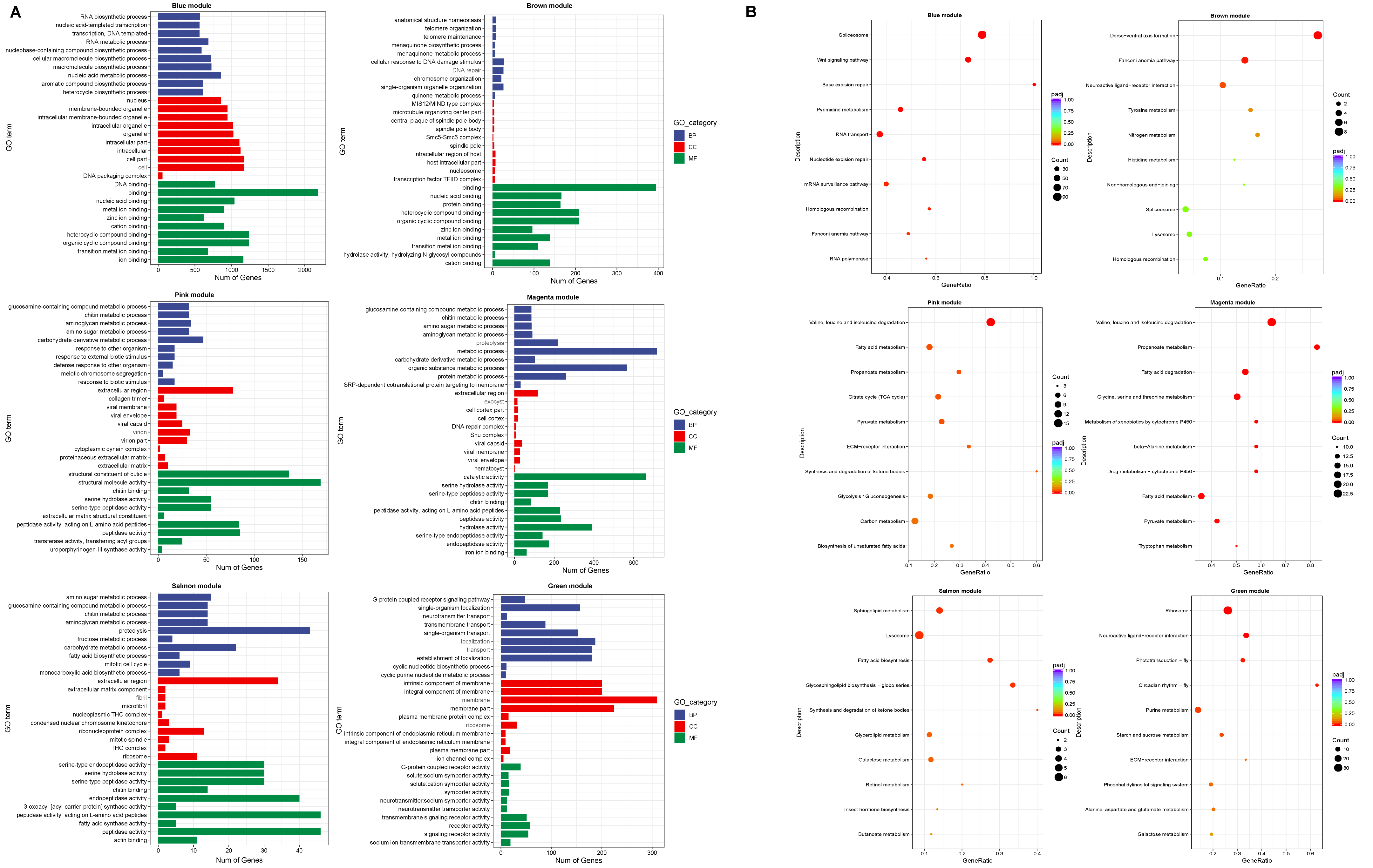

Supplement: S4 Fig — (a) Top 10 GO terms under BP (blue), CC (red) and MF (green) categories; (b) Top 10 KEGG pathways. The different colors from blue to red represent the Q value (false discovery rate value). The different sizes of the round shapes represent the number of genes in a pathway. (TIF) [file pntd.0010245.s004.tif]

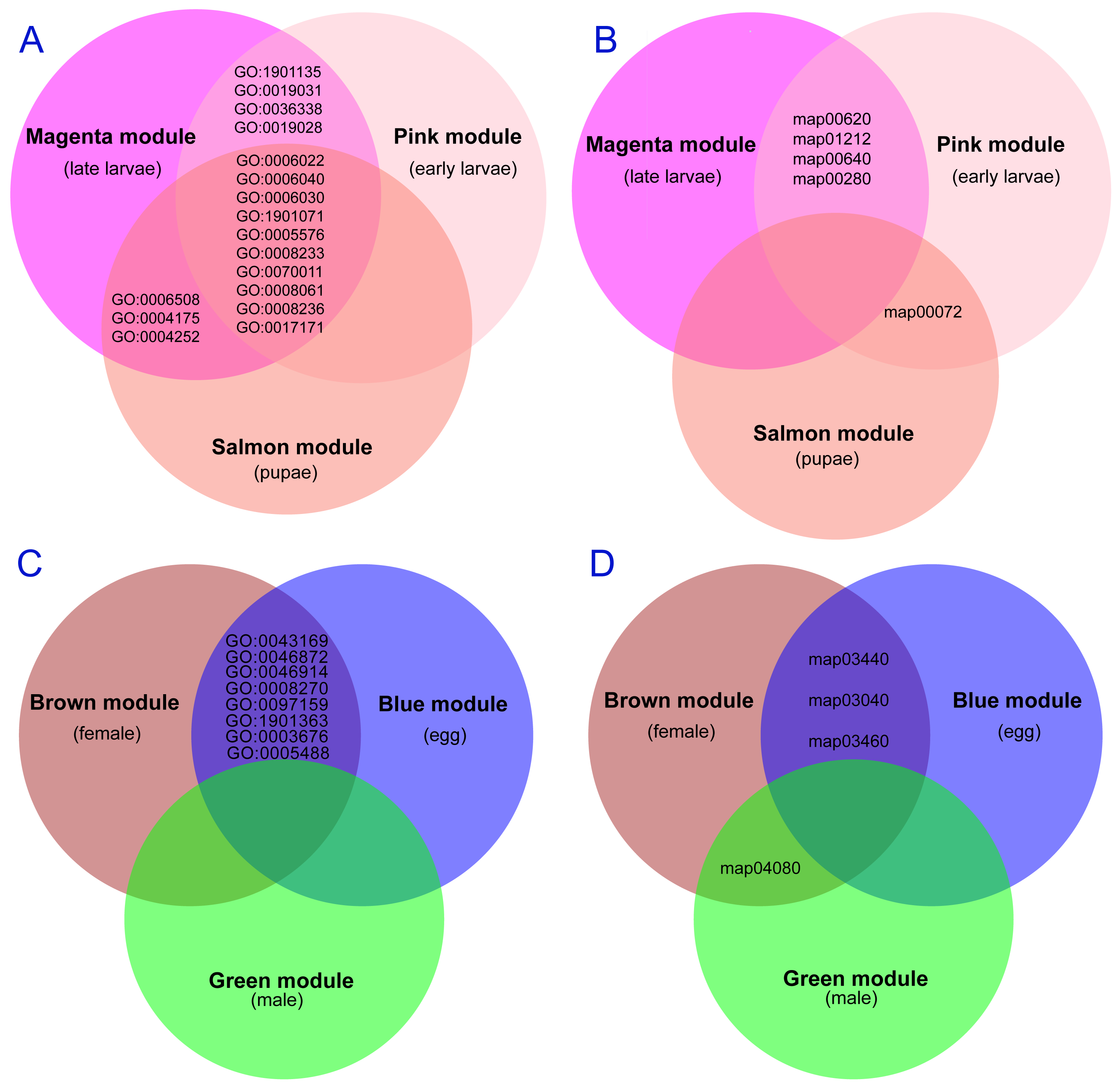

Supplement: S5 Fig — (a) co-expressed GO terms in the magenta, pink and salmon module; (b) co-expressed KEGG pathways in the magenta, pink and salmon module; (c) co-expressed GO terms in the brown, blue and green module; (d) co-expressed KEGG pathways in the brown, blue and green module. (TIF) [file pntd.0010245.s005.tif]

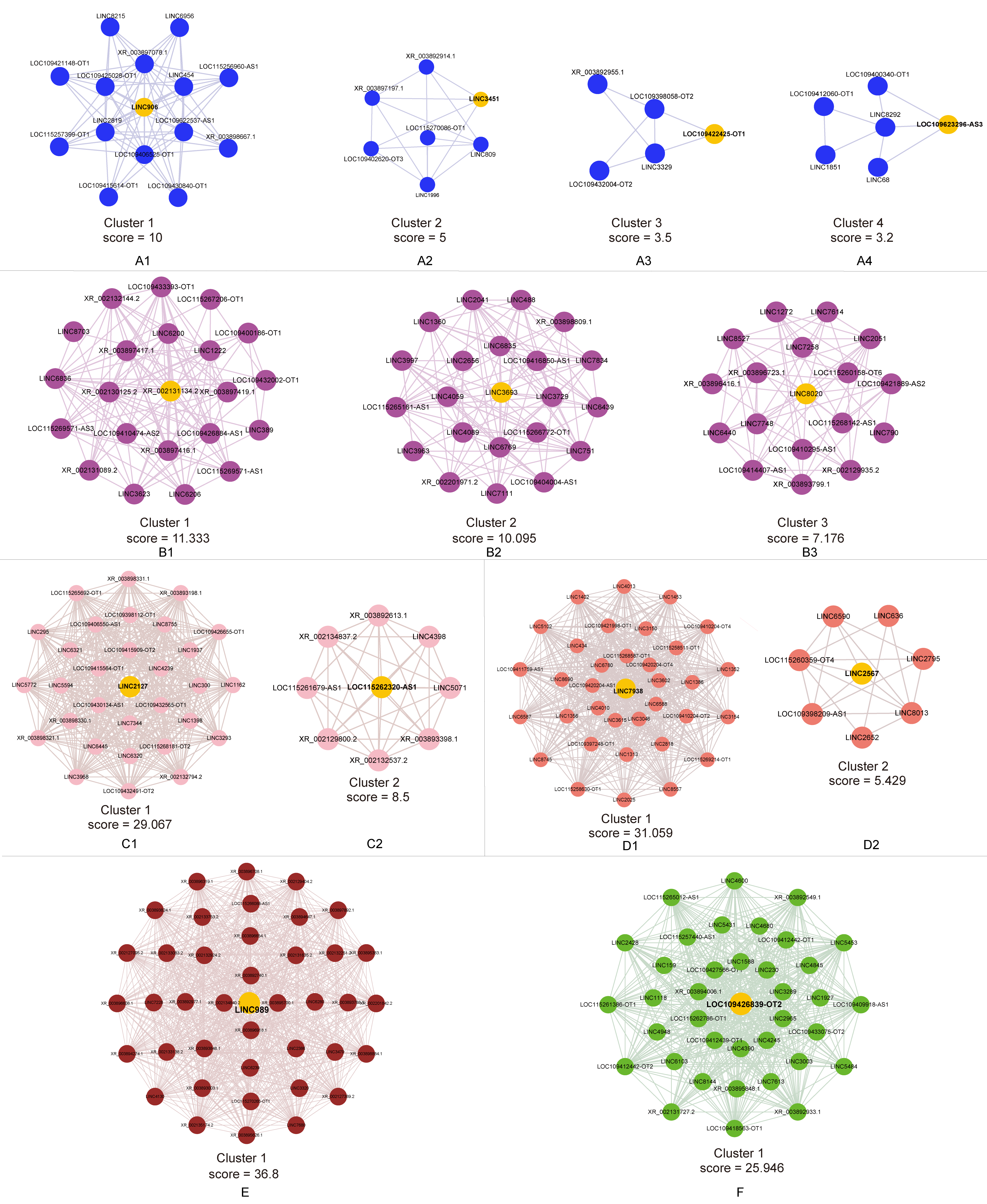

Supplement: S6 Fig — Blue module: a1-a4; magenta module: b1-b3; pink module: c1-c2; salmon module: d1-d2; brown module: e1; green module: f1. Each node represents a lncRNA, each edge denotes a target relationship between lncRNAs. (TIF) [file pntd.0010245.s006.tif]
